# Inflammatory monocytes and the pathogenesis of viral encephalitis

**DOI:** 10.1186/1742-2094-9-270

**Published:** 2012-12-17

**Authors:** Rachael L Terry, Daniel R Getts, Celine Deffrasnes, Caryn van Vreden, Iain L Campbell, Nicholas JC King

**Affiliations:** 1 Department of Pathology, School of Medical Sciences, Blackburn Circuit, The University of Sydney, Sydney, 2006, Australia; 2 Bosch Institute, The University of Sydney, Sydney, 2006, Australia; 3 School of Molecular and Microbial Biosciences, Butlin Avenue, The University of Sydney, Sydney, 2006, Australia; 4 Department of Microbiology-Immunology, Feinberg School of Medicine, Chicago Avenue, Northwestern University, Chicago, 60611, USA

**Keywords:** Ly6C^hi^ inflammatory monocytes, Viral encephalitis, Neurotrophic virus, CCL2, CCR2, VLA-4, LFA-1, Integrins

## Abstract

Monocytes are a heterogeneous population of bone marrow-derived cells that are recruited to sites of infection and inflammation in many models of human diseases, including those of the central nervous system (CNS). Ly6C^hi^/CCR2^hi^ inflammatory monocytes have been identified as the circulating precursors of brain macrophages, dendritic cells and arguably microglia in experimental autoimmune encephalomyelitis; Alzheimer’s disease; stroke; and more recently in CNS infection caused by Herpes simplex virus, murine hepatitis virus, Theiler’s murine encephalomyelitis virus, Japanese encephalitis virus and West Nile virus. The precise differentiation pathways and functions of inflammatory monocyte-derived populations in the inflamed CNS remains a contentious issue, especially in regard to the existence of monocyte-derived microglia. Furthermore, the contributions of monocyte-derived subsets to viral clearance and immunopathology are not well-defined. Thus, understanding the pathways through which inflammatory monocytes migrate to the brain and their functional capacity within the CNS is critical to inform future therapeutic strategies. This review discusses some of the key aspects of inflammatory monocyte trafficking to the brain and addresses the role of these cells in viral encephalitis.

## Background

Virus infection of the brain can cause severe and life-threatening disease. Despite this, few therapies beyond intensive supportive care are available to treat patients with encephalitis [1,2]. Anti-viral drugs have been developed for some viruses that can infect the brain, such as Herpes simplex virus (HSV)-1 and 2, and human immunodeficiency virus (HIV), but even with these treatments outcomes remain relatively poor [2-5]. Many patients succumb to disease, and survivors often suffer permanent neurological sequelae [6-9].

While the development and clinical implementation of novel anti-viral drugs may improve patient outcomes, it is becoming increasingly clear that therapies targeting pathogenic elements of the host immune response may be critical for successful intervention during infection [10-14]. Monocyte infiltration is a hallmark of central nervous system (CNS) inflammation, including viral infection. These cells migrate into the infected brain, where they differentiate into dendritic cell (DC), macrophage and, arguably, microglial populations. Once differentiated, these cells engage in a number of potent effector functions including antigen presentation and T cell stimulation, the production and secretion of numerous pro-inflammatory mediators as well as reactive oxygen species (ROS), all of which are focused on viral containment and clearance (Table 1). However, unbalanced and poorly controlled migration and effector functions of these cells may result in immune-mediated pathology, resulting in tissue damage and destruction during some infections (Table 1). Therefore, it is of high importance to understand the processes driving monocyte development, recruitment, differentiation and function, to aid in the development of novel therapeutics that inhibit immunopathological responses.

**Table 1:** Evidence for macrophage-driven pathogenesis and control of viral encephalitis

**Macrophage-derived mediators**		**Pathogenic and anti-viral functions in the central nervous system**	**Pathogenic role in mouse models**	**Anti-viral role in mouse models**
**Pro-inflammatory cytokines**	**IL-1β**	↑ pro-inflammatory cytokines	IL-1β^−/−^ mice resistant to fatal neurovirulent Sindbis virus encephalitis [15]	IL-1β^−/−^ mice exhibit increased mortality and virus loads in HSV-1 encephalitis [16]
		↑ leukocyte chemoattractants		
	**IL-6**	↑ adhesion molecules	IL-6^−/−^ mice exhibit reduced seizures in TMEV encephalitis [17]	
		↑ NO/reactive oxygen species production		
		↑ neuronal misfiring/ seizures		
		↑ neuronal		
		↑ breakdown of BBB		
		↑ MMP		
		Reviewed in [18-22]		
	**IL-12**		IL-12^−/−^ mice show decreased clinical score during MHV encephalitis [23]	Infusion of IL-12 reduces viral loads and improves survival during vesicular stomatitis virus encephalitis [24]
	**TNF**		TNF-R^−/−^ mice show improved survival in rabies virus encephalitis [25]	TNF^−/−^ mice exhibit increased mortality and virus loads in HSV-1 encephalitis [16]
**Free radicals**	**NO/reactive oxygen species**	↑ neuronal misfiring/ seizures	Inhibition of NOS2 prolonged survival in rabies virus encephalitis by delaying virus replication and inhibiting of apoptosis [26]	NOS2^−/−^ mice show increased susceptibility to CNS invasion and death in Murray Valley virus encephalitis [27]
		↑ neuronal damage/death		
		↑ formation of reactive oxygen species		
				Inhibition of NOS2 reduces mortality during Junin virus encephalitis [28] and neurovirulent Sindbis virus encephalitis [29]
			Inhibition of NOS2 prolonged survival of WNV-infected animals [30]	
		Reviewed in [31]		
**Proteases**	**MMP**	↑ breakdown of the BBB	MMP-9^−/−^ mice show reduced viral loads and increased survival during WNV encephalitis [32]	
		↑ neuronal damage/death		
		↑ demyelination		
		↑ pro-inflammatory cytokines		
		Reviewed in [33,34]		
**Neurotransmitters**	**Glutamate**	↑ neuronal misfiring/seizures	Competitive and non-competitive glutamate receptor antagonists promote survival during neurovirulent Sindbis virus encephalitis [35,36] and improved outcomes during coronavirus encephalitis [37]	
		↑ neuronal damage/death		
		↑ production of NO/ROS		
		Reviewed in [38]		

*BBB* blood brain barrier; *CNS* central nervous system; *HSV* herpes simplex virus; *MDP* macrophage/dendritic cell precursor; *MHV* murine hepatitis virus; *MMP* matrix metalloproteinases; *NO* nitric oxide; *NOS2* nitric oxide synthase-2; *ROS* reactive oxygen species; *TMEV* Theiler’s murine encephalomyelitis virus; *WNV* West Nile virus.

### Monocytes are derived from hematopoietic precursors in the bone marrow

**Figure 1:**
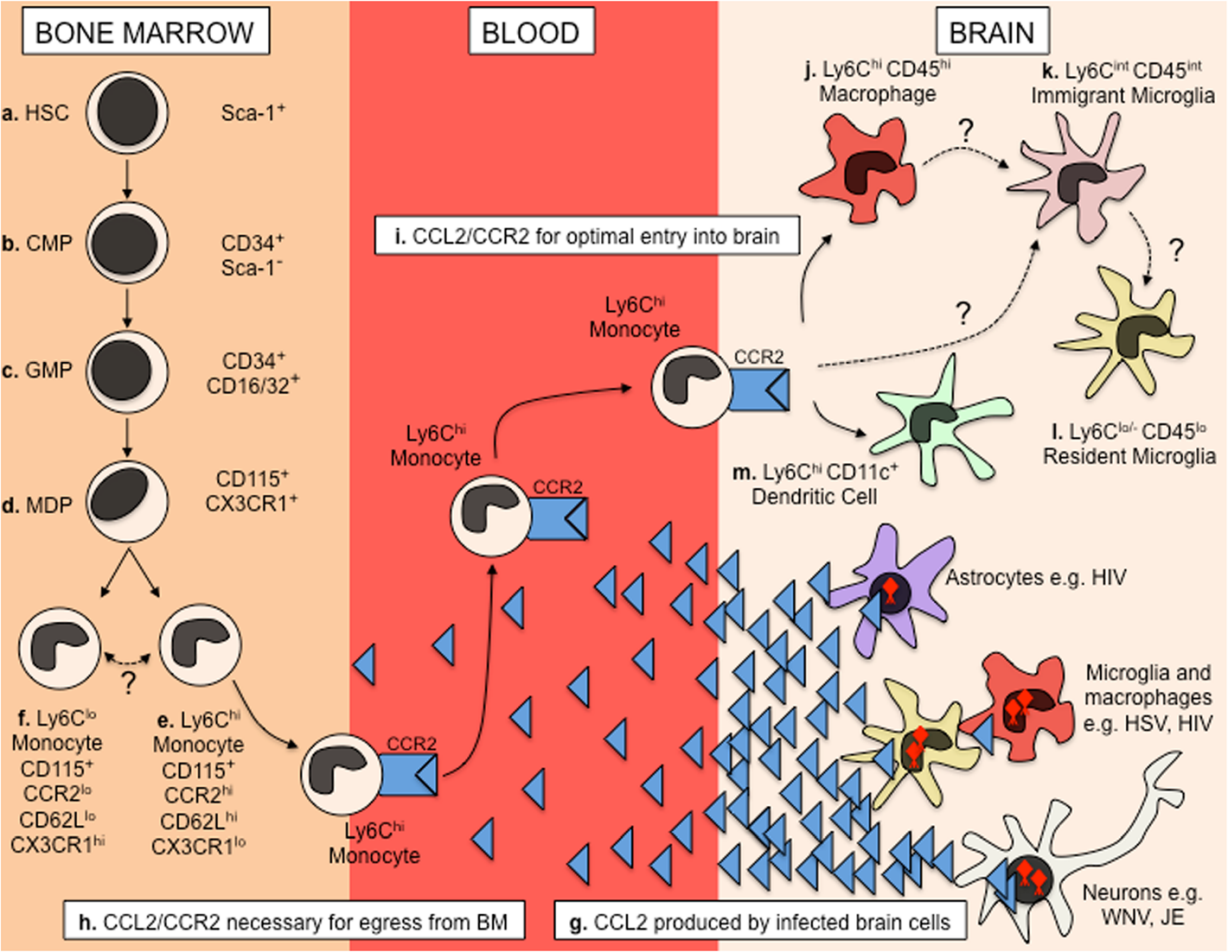
**Development of monocytes in the bone marrow and recruitment to the virus-infected brain.** Monocytes are generated from hematopoietic precursors in the bone marrow (BM). Sca-1^+^ Lin^-^ HSC **(a)** give rise to CD34^+^, Sca-1^-^ CMP **(b)**. These cells in turn give rise to a pool of precursors known as granulocyte/macrophage precursors (GMPs), which express CD34 and CD16/32 **(c)**. A fraction of these progenitors also express CD115 and CX_3_CR1 and are known as macrophage/dendritic cell precursor (MDP) **(d)**. MDPs are the direct precursors of Ly6C^hi^ inflammatory monocytes **(e)**. MDPs also give rise to circulating Ly6C^lo/-^ monocytes directly, or via a Ly6C^hi^ monocyte intermediate **(f)**. During viral encephalitis, large quantities of the chemokine CCL2 is produced by infected astrocytes, macrophages/microglia and/or neurons **(g)**. CCL2 binds the chemokine receptor CCR2, expressed at high levels by Ly6C^hi^ inflammatory monocytes, which promotes the egress of these cells from the BM **(h)** into the blood, and thus recruitment from the blood into the infected central nervous system (CNS) **(i)**. Here, these cells can give rise to CD45^hi^ Ly6C^hi^ macrophages **(j)** and/or CD45^int^ Ly6C^int^ immigrant microglia **(k)**, although it is unclear whether Ly6C^int^ immigrant microglia are derived from a Ly6C^hi^ macrophage intermediate or directly differentiate from Ly6C^hi^ monocytes. Furthermore, it is unclear whether recruited macrophages and immigrant microglia give rise to CD45^lo^ Ly6C^lo/-^ resident microglia **(l)** if/when virus is cleared from the CNS. In some models of viral encephalitis, Ly6C^hi^ inflammatory monocytes can also give rise to Ly6C^hi^/CD11c^+^ DC in the brain **(m).**

Monocytes are derived from hematopoietic stem cells (HSC) in the bone marrow (BM) (Figure 1). The earliest defined precursor is the common myeloid precursor (CMP), distinguished from HSC by the expression of CD34 but not SCA-1 [39-42] (Figure 1). These cells give rise to a pool of precursors called granulocyte/macrophage precursors (GMPs), which express CD16/32 [39]. Included within this subset is the recently defined macrophage/DC precursor (MDP), which specifically expresses high levels of the PU.1-controlled chemokine receptor CD115 (CSF-1R/M-CSFR), chemokine receptor CX_3_CR_1_ (fractalkine receptor), and Flt-3 (CD135/Flk2) [43-48] (Figure 1). The MDP gives rise to CD11b^+^, CD115^+^, F4/80^+^, CD11c^-^, Ly6G^-^ monocytes, that can be isolated from the BM and blood [49-52] (Figure 1). The spleen has also been identified as an important reservoir of undifferentiated monocytes that are rapidly deployed to sites of inflammation, including the ischemic heart and brain [53-55]. Furthermore, a recent study has shown that cardiac infarction triggers a significant increase in numbers of MDPs in the spleen, which supply monocytes throughout the duration of acute inflammation [56]. Whether the spleen is a significant source of monocytes during CNS infection is yet to be determined, but presents a critical area of future investigation. It is likely that both the BM and spleen are critical for supplying monocytes to the infected CNS, particularly in cases of acute and severe infection, in which large numbers of these cells are rapidly deployed and recruited to the brain.

### Monocytes are classified into two phenotypically and functionally distinct subsets

The MDPs give rise to two phenotypically and functionally distinct subsets of monocytes [50,57]. Ly6C^hi^ monocytes are characterized by high expression of the chemokine receptor CCR2, adhesion molecule CD62L and low expression of the fractalkine receptor CX_3_CR_1_[48,51,58]. These cells have been termed ‘inflammatory’ because they are selectively recruited to sites of inflammation and infection in many models of disease, including atherosclerosis [59-62]; rheumatoid arthritis [63]; experimental colitis [64]; cardiac infarction [65]; and CNS infections including experimental autoimmune encephalomyelitis (EAE) [66,67], amyotrophic lateral sclerosis [68], and stroke [53]. Recent studies have shown that these cells are also recruited to the virus-infected brain in animal models of HSV, HIV, murine hepatitis virus (MHV), Theiler’s murine encephalomyelitis virus (TMEV) and a number of flaviviral encephalitides, where they give rise to macrophage, DC and, arguably, to microglial populations [11,13,14,69].

Conversely, Ly6C^lo/-^ monocytes are smaller in size than their Ly6C^hi^ counterparts and express low levels of CCR2 and CD62L and high levels of CX_3_CR_1_[48,51,58] (Figure 1). Several studies have shown that Ly6C^hi^ monocytes can give rise to circulating Ly6C^lo/-^ monocytes [58,70-72]. Interest in this subset has increased substantially in the past few years [72,73]. Recent studies have described the patrolling behavior of these cells in the vasculature [73], and have shown that in some models of disease they rapidly enter inflamed tissue and can contribute to early inflammatory responses before domination by Ly6C^hi^ monocytes [73]. In the resolution phase of some diseases, Ly6C^lo/-^ monocytes are critical for wound healing and angiogenesis [50]. While apparently important in the periphery, the role of Ly6C^lo/-^ monocytes during CNS infection remains poorly defined, with little evidence supporting their migration into the brain during inflammation [74].

### Monocyte egress from the bone marrow is controlled by chemokine/chemokine receptor interactions

The importance of monocyte-derived cells in the pathogenesis of brain infection highlights the importance of understanding the pathway(s) through which monocytes migrate from the periphery into the brain. It is apparent that this process is regulated by cytokine/chemokine and integrin/cellular adhesion molecule interactions that facilitate emigration from the BM into the blood and entry into the CNS. For example, the chemokine receptor CXCR4 and one of its ligands CXCL12 (SDF-1) directly enhance VLA-4-dependent adhesion and thereby aid in retaining immature cells in the BM. Deficiency in either molecule results in impaired myelopoiesis [75-80]. In addition to CXCR4, CCR2 and its ligands, CCL2 and CCL7 (MCP-3), are a critical requirement for Ly6C^hi^ monocyte egress from the BM into the blood. CCL2/CCR2 deficiency or blockade with antibody results in monocyte accumulation in the BM in multiple disease models, including EAE, WNV and HSV encephalitides [11,61,67,81-87].

### Monocyte recruitment into the infected brain is dependent on chemokine/chemokine receptor interactions

A number of chemokines and their receptors have been implicated in the recruitment of Ly6C^hi^ monocytes from the blood and into the brain. CCR5 is expressed by Ly6C^hi^ monocytes and is important for trafficking to sites of inflammation in some models of disease. In the brain, its ligand CCL5 (RANTES) expression is highly upregulated during infection/inflammation, including WNV, MHV, HSV and tick-borne encephalitis virus encephalitides [88-92]. Another chemokine of interest that controls the trafficking of monocytes into the brain parenchyma is SDF-1/CXCL12, in conjunction with its receptor CXCR4, expressed by monocytes [93]. In animal models of CNS inflammation including EAE [94], HIV [95] and WNV [96], there is significant upregulation of CXCL12. In EAE and WNV, CXCL12 has been shown to play an important role in retaining leukocytes in the perivascular space, thereby inhibiting infiltration into the parenchyma. Loss of this interaction resulted in the loss of perivascular cuffs and uncontrolled infiltration of CXCR4^+^ leukocytes, including monocytes, into the parenchyma. [94,96].

While it is clear that there are a multitude of soluble mediators that represent potential targets for future therapies aimed at blocking monocyte migration, the CCR2/CCL2 axis remains the most potent pathway based on the available literature. Ly6C^hi^ /CCR2^hi^ monocyte recruitment into the CNS in models of stroke [53], peripheral inflammation [97], Alzheimer’s disease (AD) [98,99] and EAE [67,74,100,101] are all dependent on CCR2/CCL2 signaling (Figure 1). In the context of viral encephalitis, the CCL2/CCR2 axis is also very important. The major producers of CCL2 appear to be different depending on the infectious agent, with microglia serving as important sources during HSV infection [16,102], neurons in the case of WNV infection [11] and astrocytes in HIV encephalitis [103]. No matter the source of CCL2, the inhibition of CCL2 can significantly reduce the infiltration of inflammatory monocyte-derived macrophages and microglia into the infected brain [11-13,69,88,102,104-108].

### Monocyte recruitment into the infected brain is dependent on integrin/adhesion molecule interactions

The focus in the last decade has been heavily on the chemokines involved in monocyte trafficking, however, cellular adhesion molecules and their integrin ligands are obviously also important. In most models of viral infection, very late antigen-4 (VLA-4) and leukocyte function-associated antigen-1 (LFA-1) are expressed by Ly6C^hi^ monocytes. In addition, their respective binding partner’s vascular cell adhesion molecule-1 (VCAM-1) and inter-cellular adhesion molecule-1 (ICAM-1) are usually upregulated on endothelium and other cell types in the inflamed brain [109-115].

The importance of VLA-4 and VCAM-1 and LFA-1 and ICAM-1 in the recruitment of Ly6C^hi^ monocytes to sites of inflammation is evident in experiments using gene knockout animals or specific blockade of these molecules. VLA-4 and VCAM-1 interactions are critical for monocyte migration to the heart in models of atherosclerosis and arterial injury [116-118] and the inflamed peritoneum [119]. VLA-4 is also critical for Ly6C^hi^ monocyte infiltration of the CNS in several models of inflammation, including EAE and spinal cord injury [97,109,120]. During viral infection of the brain, we have found that recruitment of monocytes to the CNS is also VLA-4-dependent. VLA-4 antibody neutralization significantly impairs the recruitment of Ly6C^hi^ monocytes to the infected brain, in both WNV and JEV infection ([30], CvV *et al*., unpublished observations). LFA-1 and ICAM-1 interactions are also important for monocyte recruitment to atherosclerotic plaques [121,122] and to the CNS during EAE [110]. We have shown that LFA-1 is also important for recruitment of monocytes to the WNV-infected brain, however blockade resulted in a smaller reduction in monocytes infiltration compared to VLA-4 neutralization, which suggests the differential use of adhesion molecules by Ly6C^hi^ monocyte subsets which enter the WNV-infected brain [30].

### Monocytes differentiate into macrophages and dendritic cells in the infected brain

In models of CNS diseases, such as EAE and stroke, Ly6C^hi^ monocytes have been shown to primarily differentiate into macrophage and DC populations exhibiting a M1 pro-inflammatory phenotype, which *in-vitro* effectively stimulates Th1 and Th17 responses in T cells [53,66,67,74]. Similarly, in models of viral encephalitis, Ly6C^hi^ monocytes have been shown to give rise to M1 pro-inflammatory CD45^hi^ macrophages and CD11c^+^ DC populations, which express high levels of nitric oxide (NO) and TNF during HSV, WNV, MHV, TMEV and JEV ([11-14,30,69], CvV *et al.*, unpublished observations). We have shown that these CD45^hi^ macrophages are highly effective at processing and presenting antigen and effectively stimulate T cell proliferation [30].

### Resident microglia originate from a myeloid lineage distinct to that of infiltrating monocytes

Microglia are the resident macrophage population of the brain. Similar to other tissue resident cells such as Kupffer cells of the liver and Langerhans cells of the epidermis, microglia originate from the yolk sac during embryogenesis, from a myeloid lineage that is independent of BM HSC and therefore distinct from that of BM-derived monocytes [123-125]. Microglia can be distinguished from infiltrating monocyte-derived macrophages and DC by their low to intermediate expression of CD45 and lack of Ly6C expression [11,126]. In most infections, resident microglia play functionally distinct roles from that of monocyte-derived cells. For example, during acute WNV encephalitis, resident microglia express lower levels of pro-inflammatory mediators such as NO, express lower levels of MHC-II, and show a significantly reduced capacity to process antigen and stimulate T cell proliferation compared to the highly activated infiltrating macrophages [30]. In comparison, in acute TMEV infection, resident microglia and infiltrating macrophages express similar levels of pro-inflammatory cytokines and show similar antigen processing and presentation capacity; however, in chronic stages of disease, macrophages are more efficient at stimulating T cell responses [127].

### Monocytes may serve as microglial precursors during brain infection

There is evidence to suggest that infiltrating monocytes have the capacity to give rise to microglial cells in some models of CNS inflammation, including AD, Parkinson’s disease, EAE, as well as in infectious models such as scrapie and bacterial meningitis [128-134]. These immigrant microglial cells appear to play distinct functional roles compared to their resident counterparts during disease. For example, immigrant microglia are more efficient at clearing amyloid plaques than resident microglia during AD [128,135]. However, a caveat of these studies has been in the use of irradiation to generate BM chimeras to distinguish resident microglial from BM-derived cells. There are currently no immunophenotypic markers that can definitively separate these two populations. As a result, the generation of chimeras can be used distinguish tissue resident and BM-derived populations. However, irradiation can disrupt the blood–brain barrier (BBB) and promote CCL2 production, resulting in the recruitment of monocytes to the CNS [136]. Therefore, it is difficult to conclude whether monocyte engraftment is a normal feature of disease in unperturbed animals or whether it is primarily the result of brain preconditioning by irradiation. A recent study using the parabiosis model in place of irradiated BM chimeras has shown that engraftment of monocyte-derived microglia during EAE is only a transient response [137]. The parabiosis models have also been employed to show that there is no significant engraftment of monocyte-derived microglia in facial nerve axonomy or amyotrophic lateral sclerosis [138]. Also, another recent study has compared the recruitment of monocyte-derived microglia into brain during AD, using chimeric mice generated with or without head protection during irradiation. They found that these cells do not engraft the brain of protected animals [99]. However, one major caveat of the head-protection model is the existence of BM in the skull that may be capable of reconstituting the animal. Further studies are required to definitively determine whether monocyte-derived cells can give rise to microglia and if these cells truly engraft the parenchyma and remain there if/when disease is resolved.

There are few studies that examine the recruitment of monocyte-derived microglia during viral infection of the CNS. We have shown that in WNV encephalitis, inflammatory monocytes not only give rise to CD45^hi^ macrophages in the brain, but also to a CD45^int^ subset, which is phenotypically analogous to activated resident microglia, apart from the expression of Ly6C [11,30]. Although chimeras were initially utilized to investigate this phenomenon, we further confirmed that the recruitment of these monocyte-derived cells was not the result of BBB breakdown, using methods that do not use any irradiation including bone marrow adoptive transfer studies and microparticle-based systems which track these cells with minimal perturbation of the disease system [11]. Furthermore, these cells were found to contribute to the immunopathogenesis of WNV encephalitis, as CCL2 blockade significantly reduced recruitment into the CNS and prolonged survival of lethally-infected animals [11]. Current studies in our laboratory aim to determine whether monocyte-derived microglia truly engraft the brain parenchyma during WNV encephalitis, the functional role of these cells throughout infection, and whether these cells remain in the CNS after disease is resolved.

### Monocytes contribute to viral clearance or viral burden in different models of infection

Ly6C^hi^ monocytes appear to play a paradoxical role in many disease models. For example, higher mortality rates and increased pathogen loads are seen in Toxoplasma [139,140], Listeria [83,141], Cryptococcus [142,143], Yersinia infections [144], HSV-2, [145] and coronavirus [146], as well as MHV [88] when these cells are depleted. On the other hand, Ly6C^hi^ monocytes are direct targets for pathogens such as HIV, TMEV, Listeria and Toxoplasma [12,69,147-152]. Infected monocytes can be directly responsible for the dissemination of infection in a “Trojan horse” fashion into the CNS thereby potentiating disease and increasing potential mortality [153-156].

### Monocytes significantly contribute to immunopathology during brain infection

An arguable role of monocytes during brain infection is their potential contribution to immune-mediated pathology. In several models of CNS disease, Ly6C^hi^ inflammatory monocytes cause significant damage and destruction in the brain, directly contributing to morbidity and mortality. Ly6C^hi^ monocytes contribute significantly to the pathogenesis of disease during stroke [53]. Mice with CCL2^−/−^ and CCR2^−/−^ deficiency show smaller infarcts and enhanced functional outcomes relative to wild-type controls following transient cerebral ischemia [157,158]. Similarly, in models of traumatic brain injury, CCL2^−/−^ mice showed reductions in macrophage infiltration and lesion volume compared to wild-type mice, corresponding with improved functional recovery after injury [159]. In addition, CCR2^−/−^ and CCL2^−/−^ mice exhibit milder symptoms and, in some models, are completely resistant to the development of EAE [100,136,160,161]. Furthermore, a recent study has shown that Ly6C^hi^ monocyte recruitment to the CNS is detrimental in amyotrophic lateral sclerosis [68]. In the case of encephalitic disease, studies in our laboratory using WNV as well as others using TMEV have shown that Ly6C^hi^ monocytes are recruited into the infected brain where they contribute significantly to the immunopathogenesis of disease. Inhibition of inflammatory monocyte migration into the WNV or TMEV-infected brain can significantly reduce morbidity and mortality [11,12,69,108]. Furthermore, abrogation of monocyte migration into the CNS during MHV encephalitis results in the delayed onset of demyelinating disease [105]. The precise pathways through which inflammatory monovcytes contribute to pathology are still under intense investigation. However, it is clear that differentiation into effector cells such as macrophages and DC plays a substantial role. Once differentiated, these cells are significant producers of NO, matrix metalloproteinases (MMP) and other factors known to culminate in tissue destruction, breakdown of the BBB, as well as neuronal damage (Table 1). While in many organs such toxicity is not a major concern due to regenerative capabilities, the brain is largely comprised of many irreplaceable cellular subsets. As such not only is mortality a concern, in patients that survive serious CNS inflammatory insults will often suffer long-term sequelae and neurological imbalance [6-9].

## Conclusions

Although Ly6C^hi^ monocyte infiltration is a hallmark of viral encephalitis, the role of these cells in viral clearance and immunopathology is not well defined. While it is clear that these cells are critical for the control and clearance of some viruses, they are directly responsible for recruiting others into CNS, or cause significant immunopathology. Future studies which target monocyte development and migration to the CNS in a therapeutic manner will not only provide significant insight into pathways by which monocytes are recruited to the CNS, but will identify new targets for intervention during viral encephalitis.

## Abbreviations

AD: Alzheimer’s disease; BBB: Blood–brain barrier; BM: Bone marrow; CNS: Central nervous system; CMP: Common myeloid precursor; DC: Dendritic cells; EAE: Experimental autoimmune encephalomyelitis; GMP: Granulocyte/macrophage precursor; HIV: Human immunodeficiency virus; HSC: Hematopoietic stem cells; HSV: Herpes simplex virus; ICAM-1: Inter-cellular adhesion molecule-1; JEV: Japanese encephalitis virus; LFA-1: Leukocyte function-associated antigen-1; MDP: Macrophage/DC precursor; MHV: Murine hepatitis virus; MMP: Matrix metalloproteinases; NO: Nitric oxide; NOS2: Nitric oxide synthase-2; ROS: Reactive oxygen species; TMEV: Theiler’s murine encephalomyelitis virus; VCAM-1: Vascular cell adhesion molecule-1; VLA-4: Very late antigen-4; WNV: West Nile virus.

## Competing interests

The authors declare that they have no competing interests.

## Authors’ contributions

RLT drafted the manuscript. DRG, CD, CVV, ILC and NJCK contributed to the interpretation and critical evaluation of content and revision of the manuscript. All authors read and approved the final manuscript.
